# Assessing awareness and use of HIV self-testing kits after the introduction of a community-based HIV self-testing programme among men who have sex with men in Kenya

**DOI:** 10.1371/journal.pgph.0001547

**Published:** 2023-08-18

**Authors:** Souradet Y. Shaw, Stella Leung, Shajy Isac, Helgar Musyoki, Mary Mugambi, Japheth Kioko, Janet Musimbi, Kennedy Olango, Samuel Kuria, Martin K. Ongaro, Jeffrey Walimbwa, Memory Melon, Faran Emmanuel, Stephen Moses, James F. Blanchard, Michael Pickles, Lisa Lazarus, Robert R. Lorway, Marissa L. Becker, Sharmistha Mishra, Parinita Bhattacharjee

**Affiliations:** 1 Institute for Global Public Health, Community Health Sciences, University of Manitoba, Winnipeg, Manitoba, Canada; 2 India Health Action Trust, Delhi, India; 3 National Syndemic Disease Control Council, Nairobi, Kenya; 4 Partners for Health and Development in Africa, Nairobi, Kenya; 5 Men Against AIDS Youth Group, Kisumu, Kenya; 6 Mamboleo Peer Empowerment Group, Kiambu, Kenya; 7 HIV and AIDS People’s Alliance of Kenya, Mombasa, Kenya; 8 G10 Research Advisory Committee, Nairobi, Kenya; 9 Medical Research Council Centre for Global Infectious Disease Analysis, School of Public Health, Imperial College London, London, England; 10 Institute of Medical Sciences, University of Toronto, Toronto, Canada; University of Minnesota Medical School Twin Cities, UNITED STATES

## Abstract

Men who have sex with men (MSM) bear a disproportionate burden of new HIV infections in Kenya, while experiencing discrimination, leading to suboptimal levels of HIV care. HIV self-testing (HIVST) is a tool to increase HIV screening and earlier diagnosis; however, questions remain regarding how best to scale-up HIVST to MSM in Kenya. The main objective of this study was to examine changes in knowledge and use of HIVST after implementation of a community-led HIVST project. Participants were MSM recruited from Kisumu, Mombasa, and Kiambu counties. Data were collected from two rounds (Round 1: 2019; Round 2: 2020) of serial cross-sectional integrated biological and behavioural assessments (IBBA), pre-, and post-project implementation. Two main outcomes were measured: 1) whether the respondent had ever heard of HIVST; and 2) whether they had ever used HIVST kits. Changes in outcomes between IBBA rounds were examined using modified multivariable Poisson regression models; adjusted prevalence ratios (aPR) and 95% confidence intervals (95% CI) are reported. A total of 2,328 respondents were included in main analyses. The proportion of respondents who had heard of HIVST increased from 75% in Round 1 to 94% in Round 2 (aPR: 1.2, 95% CI: 1.2–1.3), while those reporting using an HIVST kit increased from 20% to 53% (aPR: 2.3, 95% CI: 2.0–2.6). Higher levels of education and HIV programme awareness were associated with both outcomes. Awareness and use of HIVST kits increased after implementation of a community-led HIVST implementation project, demonstrating the importance of integration with existing community groups.

## Introduction

Kenya has one of the largest HIV epidemics in the world, with the National AIDS and STI Control Programme (NASCOP) of Kenya estimating adult HIV prevalence at 4.9% (or 1.3 million individuals) in 2018 [1,2]. With HIV prevalence between 19%-40% in some studies [3,4], key populations (KPs) such as men who have sex with men (MSM) bear an unequal burden of HIV infection in Kenya [5–9], and have thus been prioritized in Kenya’s national HIV response [10]. A High Court ruling in 2019 upheld the criminalization of same-sex sexual behaviours in Kenya [11], continuing stigmatization and discrimination of MSM, and limiting access to healthcare, which in turn increases the vulnerability of MSM to HIV/AIDS [12–14]. NASCOP has estimated that only 53% of MSM living with HIV were known and registered in HIV programmes as of December 2018 [15]. Screening is an important entry point for HIV prevention services [16], while also facilitating earlier diagnosis and treatment of HIV, which can lead to reductions in transmission, and HIV-related morbidity and mortality [17,18]. Screening, earlier diagnosis, and linkage to prevention and treatment have thus been critical priorities for national HIV programs [19], including those in Kenya [20]. HIV self-testing (HIVST) is a promising approach to optimize screening and earlier diagnosis of HIV among MSM [21,22]. A recent systematic review found increased testing uptake and higher test-positivity yield among MSM using HIVST kits [23]. External “system shocks” like the COVID-19 pandemic have limited access to clinic-based HIV testing, therefore highlighting the importance of having a wider range of testing options available [24].

There have been few studies examining the acceptability and effectiveness of HIVST among MSM in Kenya. In partnership with NASCOP, MSM research networks, and MSM-serving community-based organizations (CBOs) and non-governmental organizations (NGOs) in Kenya, the University of Manitoba implemented a community-based project to evaluate the effectiveness of HIVST interventions within already existing HIV prevention and treatment programmes for MSM in Kenya [3,25]. Using serial cross-sectional data from two rounds of an Integrated Biological and Behavioural Assessment (IBBA) implemented as part of the project’s *Program Science*-based evaluation plan [25], the main objective of our analyses was to examine changes in respondents’ awareness and uptake of HIVST between rounds. The secondary objective of this study was to describe HIV program exposure, conditioned on reported use of HIVST kits, by round.

## Methods

### Ethics statement

Ethics approval was obtained from the institutional review boards of the University of Nairobi (P557/08/2018) and University of Manitoba (HS22205). We followed NASCOP’s guidelines in conducting sexual and reproductive health research with adolescent key populations whereby those 15 years and above are considered mature/emancipated minors [26,27]. Given this, and the fact that HIV testing in Kenya without a guardian is 15 years and above, ethics boards in Kenya and Manitoba allowed respondents to give consent and participate in the study without guardian consent. Additional information regarding the ethical, cultural, and scientific considerations specific to inclusivity in global research is included in the Supporting Information (S1 Checklist).

### Study setting & intervention

In 2008, Kenya became one of the first countries to develop national guidelines on HIVST, and through NASCOP, has had a history of providing HIVST kits to the general population, with an official launch of an HIVST strategy in 2017 [28]. This launch included the distribution and provision of an estimated 500,000 HIVST kits to select healthcare facilities in 2017 alone, in addition to HIVST kits being made available through the private sector [28]. However, at the time of the study, NASCOP did not have a specific scale-up strategy for provision of HIVST to KPs. Given this gap, the HIVST study was co-designed with NASCOP, community researchers and CBO leaders [3], and conducted in three counties in Kenya: Kisumu, Mombasa and Kiambu, representing the western, coastal, and central regions of Kenya, respectively. The three sites were chosen because of persistently high HIV prevalence, with self-reported HIV prevalence among MSM ranging between 13%-23% in 2017 [5]; the relatively large communities of MSM in each of the counties, with NASCOP’s size estimates (including male sex workers) ranging from 1,873 (Kiambu) to 4,328 (Mombassa) men in 2019 [10]; and well-established community health infrastructure for delivering sexual health services to MSM [29]. Work on the study started in early 2019.

The intervention targeted MSM above the age of 15 years and used several service delivery mechanisms to make HIVST accessible to MSM, including distribution through facility and community settings. In Kenya, the minimum legal age for HIV testing is 15 years and over. Facility distribution included: clinics and Drop-in Centres (DICs); and outreach clinics located in hotspots. Community distribution included direct distribution through peer educators at hotspots and other gathering sites, and indirect distribution via word-of-mouth with MSM known to programs, who could then redistribute kits to peers and relatives. HIVST kits were distributed by trained personnel, who also provided information and education about sexuality, risks of unsafe sexual behaviours, HIV testing services, and prevention and treatment services. Primary contacts were given the option to choose either assisted (supported and in presence of outreach or clinical staff) or unassisted (on their own) self-testing, depending on their preference. In addition, demand generation for HIVST was conducted at physical locations and through virtual media such as Facebook and WhatsApp groups, as well as Kenya’s Ministry of Health HIV testing website and the National HIV Testing Helpline. The intervention was introduced after the first round of data collection of the IBBA (described below).

### Study design and participants

We used data from two rounds of serial cross-sectional IBBA surveys conducted among MSM recruited from physical and virtual sites in the three counties [3]. Physical sites included physical locations such as bars, streets, and sex dens, while virtual sites included web-based apps and social network sites. Data collection took place from May to July 2019 (Round 1), and from August to October 2020 (Round 2). Participants were included if they: (a) identified as male; (b) reported engaging in anal or oral sex with another male in the previous 12 months; and (c) were of 15 years of age or above. A multi-stage cluster sampling approach involving physical and virtual sites was used to recruit 1200 participants (400 in each county) for each round. The methodology is described in detail elsewhere [3]. Briefly, a sampling frame was generated using programmatic mapping and size estimation of physical and virtual sites [10,30,31]. Sites were sampled to recruit 200 MSM each from physical and virtual sites in each county. Recruitment involved random sampling of virtual and physical sites; for virtual sites, peer researchers used each randomly selected virtual site to further randomly recruit the pre-defined number of potential participants who were online when the peer researcher logged into the site. Respondents from both physical and virtual sites provided a list of known contacts that identify as MSM, from which a random sample of one contact was selected for recruitment into the study.

### Data collection

Data collection took place in private spaces (e.g. CBOs, drop in centres, and clinics), at a time and location convenient to the participant. Eligible individuals from virtual and physical sites were requested to visit a specified data collection site, where they were invited to provide informed, written consent; participants were informed they could choose to participate in all or some elements of the IBBA. Trained researchers administered a face-to-face structured questionnaire in Kiswahili or English. All participants were offered HIV testing and counselling with a rapid two-test algorithm as per Kenya national guidelines, with onsite reporting of results. If their HIV test was positive, participants were offered accompanied referral to an MSM-focused clinic, or to a government testing and treatment clinic. All participants were provided with condoms and lubricants and information on HIV self-testing. Those who were seronegative were offered HIV pre-exposure prophylaxis within the clinics. Participants were asked to provide a dried blood spot for HIV confirmatory serology, performed at the HIV National Laboratory in Nairobi, using the Bioelisa HIV test kit for screening and if positive, the Murex HIV1-2-O test for confirmation. Completed questionnaires were transferred to Nairobi and data were entered into an electronic database (CSPro, US Census Bureau and ICF International). The data collection process is detailed further in the study protocol paper [25].

### Measurements

**Fig 1:**
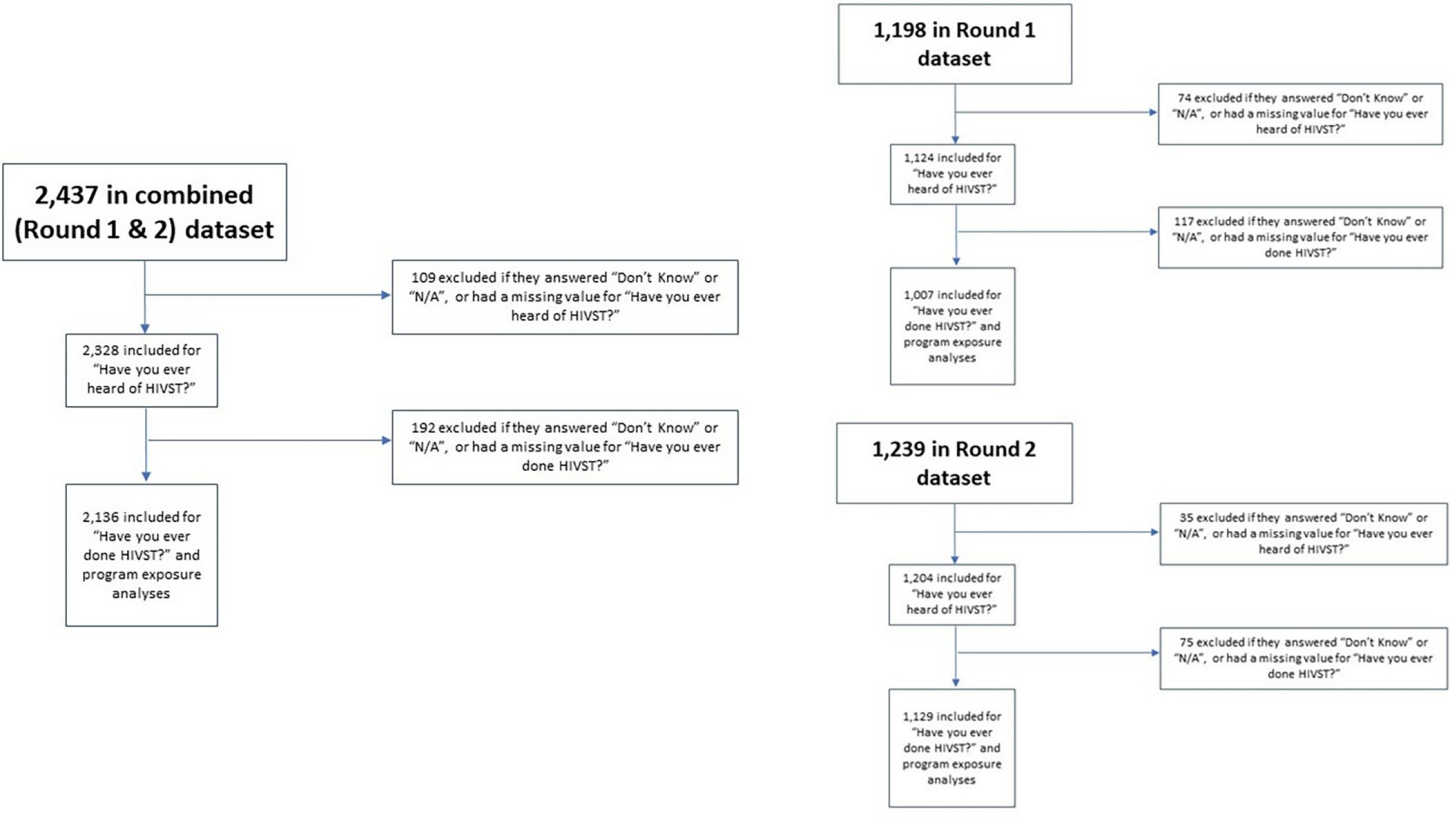
Study exclusion flow diagram.

For the main analyses, the two main outcomes were based on the following questions: 1) “Have you ever heard of HIV self-testing?”; and 2) “Have you ever done self-testing?”. Whether respondents were from Round 1 or 2 was the main exposure variable. Only respondents who answered “Yes” or “No” to “Have you ever heard of HIV self-testing?” (i.e., those who answered “Don’t know”, “N/A”, or whose response was missing) were included in analyses of the first outcome, as many respondents who did not have a response for this first outcome variable also did not have responses for important variables such as age and education. Because of skip patterns in the survey tool, only those who answered “Yes” or “No” to “Have you ever heard of HIV self-testing?” were able to answer the follow-up question on HIVST use. For these analyses, only those who responded “Yes” or “No” to this second question were included, for similar reasons; thus, sample size differed between analyses. Fig 1 contains a flow diagram of study exclusions.

Guided by the HIV literature, we included socio-demographic, sexual behaviour, and engagement with HIV services characteristics, measured at the time of project participation, as potential confounders [3,4,32–34]. Socio-demographic characteristics included current age, highest level of educational attainment, and monthly income. Variables related to sexual behaviour included: preferred sexual position/role, age at first anal/oral sex with a man, number of different male partners in the past one month, receipt of money or gifts in exchange for sex with a man (ever), condom use at last sex with a male partner, and whether the participant preferred to meet their partners in physical sites, virtual sites, or both [3]. The following question was used to define the three groups: “which are the different places/locations through which you have met other male sexual partners?”. Respondents were allowed to choose multiple responses and categorized according to where they met their partners [3]. Two measures of engagement with HIV services were used: contact by a peer educator in the prior three months; and visit to a MSM -focused clinic/drop-in centre in the previous three months.

For the secondary analyses, and to explore program exposure further, we compared program exposure between men who used HIVST kits and those who did not, in both Rounds 1 and 2. Program exposure was conceptualized as “Contact/Visit Exposure” and “Services Exposure”. The following questions were used to explore Contact/Visit Exposure: “Have you visited a clinic or drop-in Centre that provides health information or services to MSM in the past three months?”; “Have you been contacted by a peer educator/outreach worker in the past three months?”; and “The last time when you were contacted by a peer educator/outreach worker, how/where did he contact you?”. Respondents were allowed a single response for *Contact/Visit Exposure* questions. *Services Exposure* was explored using the following two variables: “The last time when you were contacted by a peer educator/outreach worker, what services did you receive?”, and “The last time you visited a clinic/drop-in-centre, what service/s did you receive?”. For the questions on services received, respondents were allowed multiple responses from a pre-defined list, which also included an option for “Other”.

### Statistical analyses

For the primary analyses, we used χ^2^ tests for comparison of proportions, and the Kruskal–Wallis test to compare medians. Change in the main outcome variables (awareness and use of HIVST) between the two rounds of the IBBA was quantified through the use of crude and fully-adjusted prevalence ratios (PRs) and 95% confidence intervals (95%CI) estimated by a modified Poisson regression approach using separate generalized linear regression models with robust standard errors specified [35]. Fully-adjusted models were constructed using a combination of *a priori* specified variables, and variables that were significant at the p < .10 level in partially-adjusted analyses (models with the main explanatory variable and each potential confounding variable), using the likelihood ratio test. The following variables were of interest *a priori*: age, county, level of education, number of partners in the last month, and solicitation type. In the secondary analyses, program exposure was compared between those reporting use of HIVST kits and those who did not, by round. Data were analysed using SAS 9.4 (SAS Institute Inc., Cary, NC); the GENMOD procedure was used in all regression analyses.

## Results

From an original sample size of 2,437, approximately 4% (n = 109) were excluded as they did not respond “Yes” or “No” to the question “Have you ever heard of HIV self-testing?”, leaving a sample size of 2,328 for the primary analyses (Round 1: 1,124; Round 2: 1,204). For the question “Have you ever taken an HIV self-test?”, a further 192 respondents were excluded, for a sample size of 2,136 (Round 1: 1,007; Round 2: 1,129). Table 1 shows a comparison of selected characteristics from respondents by round. More men reported ever hearing about HIVST (94% vs. 75%, p < .0001) and using HIVST kits (53% vs. 20%, p < .0001) in Round 2, compared to Round 1. Most participants met their partners in both physical and virtual locations, with Round 2 including a higher proportion of those who did so (63% vs. 68%, p = .035). Round 2 had a higher proportion of participants with post-secondary education (46% vs. 32%, p < .0001), more men reporting being contacted by a peer educator or outreach worker in the last three months (70% vs. 50%, p < .0001), and visiting an MSM-focused clinic or drop-in centre in the last three months (63% vs. 47%, p < .0001).

**Table 1:** Selected characteristics, men who have sex with men enrolled in HIV self-test study Kenya, by survey round (N = 2,328).

	**Round 1**	**Round 2**	**p-value**
	**N = 1,124**	**N = 1,204**	
**Places Where Male Partners Met, n(%)** ^**a**^			
Both physical and virtual sites	710 (63.2)	817 (67.9)	0.0347
Physical sites	160 (14.2)	136 (11.3)	
Virtual sites	254 (22.6)	251 (20.9)	
**County, n(%)**			
Kiambu	382 (34.0)	409 (34.0)	
Kisumu	378 (33.6)	399 (33.1)	
Mombasa	364 (32.4)	396 (32.9)	0.957
**Age (years)**			
Median (IQR)	23.0 (21.0–27.0)	24.0 (21.0–27.0)	0.369
<25 Years, n(%)	688 (61.2)	717 (59.6)	0.421
25+ Years, n(%)	436 (38.8)	487 (40.5)	
**Highest Level of Education, n(%)**			
Up to Primary	219 (19.5)	128 (10.6)	<0.0001
Secondary	551 (49.0)	521 (43.3)	
Post-Secondary	354 (31.5)	555 (46.1)	
**Monthly Income**			
Median (IQR)	8000.0 (0.0–15000.0)	8000.0 (0.00–15000.0)	0.555
No Income, n(%)	340 (30.3)	321 (26.7)	0.021
<10000 Shilling/<100 USD, n(%)	261 (23.2)	336 (27.9)	
10000+ Shilling/100+ USD, n(%)	523 (46.5)	547 (45.4)	
**Sexual Position Preference, n(%)**			
Predominantly receptive (bottom)	248 (22.1)	253 (21.0)	0.767
Predominantly insertive(top)	520 (46.3)	556 (46.2)	
Both receptive and insertive	356 (31. 7)	395 (32.8)	
**Age at first anal/oral sex with a man (years)**			
Median (IQR)	18.0 (16.0–20.0)	18.00 (16.0–20.0)	0.090
<15 Years, n(%)	117 (10.4)	113 (9.4)	0.054
15 to 17 Years, n(%)	399 (35.5)	380 (31. 6)	
18+ Years, n(%)	608 (54.1)	711 (59.1)	
**Number of different male sexual partners in the past one month**			
Median (IQR)	2.0 (1.0–3.0)	2.0 (1.0–3.0)	0.872
<2	470 (41.8)	567 (47.1)	0.011
2+	654 (58.2)	637 (52.9)	
**Received money/gift in exchange of sex with man, n(%)**	680 (60.5)	709 (58.9)	0.447
**Condom use with last male sexual partner, n(%)**	800 (71.2)	787 (65.4)	0.0028
**Ever heard of HIV self-testing, n(%)**	843 (75.0)	1134 (94.2)	<0.0001
Ever done HIV Self-Testing, n(%)* (R1: 1,007; R2: 1,129)	204 (20.3)	602 (53.3)	<0.0001
**Contacted by a peer educator/outreach worker in the last 3 months**	559 (49.7)	842 (69.9)	<0.0001
**Visited an MSM clinic/drop-in centre in the past 3 months**	529 (47.1)	761 (63.2)	<0.0001
**Ever been tested for HIV**	1093 (97.2)	1183 (98.3)	0.122

^a^Physical sites include hotspots, nightclubs, etc.; Virtual sites include social media, dating apps, etc.

Table 2 shows the association between selected characteristics and having ever heard of HIVST. Round 2 respondents were more likely to report hearing about HIVST (PR: 1.26, 95% CI: 1.21–1.30), as did those who met their male partners at virtual sites only (88%), and respondents from Kisumu (93%). Ever hearing of HIVST was strongly associated with education; 65% of those reporting “up to primary” level of education had heard of HIVST, while this proportion was 85% and 93% among those reporting “secondary”, and “post-secondary” levels of schooling, respectively. Those reporting being contacted by a peer/outreach worker in the last three months (91% vs. 76%; PR: 1.21, 95% CI: 1.16–1.26) were more likely to report ever hearing of HIVST. Participants with 2+ partners in the past month (83% vs. 88%; PR: 0.94, 95% CI: 0.91–0.98), and those reporting ever receiving money in exchange for sex (83% vs. 88%; PR: 0.94, 95% CI: 0.91–0.97) were less likely to report ever hearing about HIVST. In fully adjusted models, Round 2 participants were more likely to report having ever heard of HIVST (aPR: 1.21, 95% CI: 1.17–1.25).

**Table 2:** Crude and fully-adjusted prevalence ratios (PR) and 95% confidence intervals (95%CI) of selected characteristics and their association with ever hearing of HIV self-testing kits, from the Kenya HIV self-testing study (N = 2,328).

	Have you heard of HIV self-testing? (N: 2,328)		Crude PR (95% CI)	Fully-adjusted PR (95% CI)
	Yes (No., %)	No (No., %)		
Round (row %)^*b*^				
Round 1	843 (75.0)	281 (25.0)	*Ref*	*Ref*
Round 2	1134 (94.2)	70 (5.8)	1.26 (1.21–1.30)	1.21 (1.17–1.25)
Places Where Male Partners Met^a^^,^ ^*b*^				
Both physical and virtual sites	1306 (85.5)	221 (14.5)	*Ref*	*Ref*
Physical sites	228 (77.0)	68 (23.0)	0.90 (0.84–0.96)	0.94 (0.89–1.00)
Virtual sites	443 (87.7)	62 (12.3)	1.03 (0.99–1.07)	1.03 (0.99–1.08)
County^*b*^				
Kiambu	694 (87.7)	97 (12.3)	*Ref*	*Ref*
Kisumu	724 (93.2)	53 (6.8)	1.06 (1.03–1.10)	1.11 (1.07–1.15)
Mombasa	559 (73.6)	201 (26.5)	0.84 (0.80–0.88)	0.93 (0.88–0.98)
Age^*b*^				
<25 years	1203 (85.6)	202 (14.4)	*Ref*	*Ref*
25+ years	774 (83.9)	149 (16.1)	0.98 (0.94–1.01)	1.02 (0.99–1.06)
Highest Level of Education^*b*^				
Up to Primary	226 (65.1)	121 (34.9)	*Ref*	*Ref*
Secondary	910 (84.9)	162 (15.1)	1.30 (1.20–1.41)	1.20 (1.11–1.29)
Post-Secondary	841 (92.5)	68 (7.5)	1.42 (1.31–1.54)	1.24 (1.15–1.34)
Monthly Income				
No income	569 (86.1)	92 (13.9)	*Ref*	--
<10000 Shilling	503 (84.3)	94 (15.8)	0.98 (0.94–1.03)	
10000+ Shilling	905 (84.6)	165 (15.4)	0.98 (0.94–1.02)	
Sexual Position Preference				
Predominantly insertive	913 (84.9)	163 (15.2)	*Ref*	--
Predominantly receptive	421 (84.0)	80 (16.0)	0.99 (0.95–1.04)	
Receptive and insertive	643 (85.6)	108 (14.4)	1.01 (0.97–1.05)	
Age at first anal/oral sex with a man				
<15 Years	198 (86.1)	32 (13.9)	*Ref*	
15 to 17 Years	662 (85.0)	117 (15.0)	0.99 (0.93–1.05)	
18+ Years	1117 (84.7)	202 (15.3)	0.98 (0.93–1.04)	
Number of different male sex partners (past 1 month)^*b*^				--
<2	909 (87.7)	128 (12.3)	*Ref*	*Ref*
2+	1068 (82.7)	223 (17.3)	0.94 (0.91–0.98)	0.99 (0.96–1.03)
Received money/gifts for sex (ever)^c^				
No	827 (88.1)	112 (11.9)	*Ref*	*Ref*
Yes	1150 (82.8)	239 (17.2)	0.94 (0.91–0.97)	0.97 (0.94–1.00)
Condom use with last male sex partner^c^				
No	616 (83.1)	125 (16.9)	*Ref*	*Ref*
Yes	1361 (85.8)	226 (14.2)	1.03 (0.99–1.07)	1.03 (1.00–1.07)
Contacted by peer/outreach worker in last 3 months^c^				
No	700 (75.5)	227 (24.5)	*Ref*	*Ref*
Yes	1277 (91.2)	124 (8.9)	1.21 (1.16–1.26)	1.12 (1.08–1.16)

^a^Physical sites include hotspots, nightclubs, etc.; Virtual sites include social media, dating apps, etc.

^*b*^*a priori* chosen for fully-adjusted regression model.

^c^p < .10 in partial regression model (including Round 2 vs. 1 variable and present variable).

A total of 2,136 participants were included in analyses examining use of HIVST kits (Table 3). At 40%, respondents who met their partners in both physical and virtual locations were most likely to report using kits; and similar to the first outcome, respondents from Kisumu were most likely to report using an HIVST kit, at 47%, while a strong association between education and reported use of HIVST kits was again demonstrated. Of some interest, those reporting 2+ different sex partners in the past 1 month, at 35%, were less likely to report HIVST kit use, compared to those reporting just one sex partner in the past one month (41%; PR: 0.86, 95% CI: 0.77–0.96). In fully-adjusted models, Round 2 participants were more likely to report having ever used an HIVST kit (aPR: 2.30, 95%CI: 2.01–2.63). Except for those reporting “post-secondary” education and the “ever heard of HIVST” outcome, increases between rounds were seen across all strata for both outcomes (S1 Table).

**Table 3:** Crude and fully-adjusted prevalence ratios (PR) and 95% confidence intervals (95%CI) of selected characteristics and their association with ever using an HIV self-test kit from the Kenya HIV self-testing study (N = 2,136).

	Have you taken an HIV self-test? (N: 2,136)		Crude PR (95% CI)	Fully-adjusted PR (95% CI)
	Yes (No., %)	No (No., %)		
Round (row %)^*b*^				
Round 1	204 (20.3)	803 (79.7)	*Ref*	*Ref*
Round 2	602 (53.3)	527 (46.7)	2.63 (2.30–3.01)	2.30 (2.01–2.63)
Places Where Male Partners Met^a^^,^^*b*^				
Both physical and virtual sites	565 (40.4)	835 (59.6)	*Ref*	*Ref*
Physical sites	75 (29.4)	180 (70.6)	0.73 (0.60–0.89)	0.79 (0.65–0.95)
Virtual sites	166 (34.5)	315 (65.5)	0.85 (0.74–0.98)	1.01 (0.88–1.16)
County^*b*^				
Kiambu	246 (32.5)	511 (67.5)	*Ref*	*Ref*
Kisumu	355 (47.4)	394 (52.6)	1.46 (1.28–1.66)	1.51 (1.33–1.72)
Mombasa	205 (32.5)	425 (67.5)	1.00 (0.86–1.17)	1.16 (1.00–1.34)
Age^*b*^				
<25 years	478 (36.7)	825 (63.3)	*Ref*	*Ref*
25+ years	328 (39.4)	505 (60.6)	1.07 (0.96–1.20)	1.11 (1.00–1.23)
Highest Level of Education^*b*^				
Up to Primary	62 (22.1)	218 (77.9)	*Ref*	*Ref*
Secondary	326 (33.0)	662 (67.0)	1.49 (1.18–1.89)	1.29 (1.02–1.62)
Post-Secondary	418 (48.2)	450 (51.8)	2.17 (1.73–2.74)	1.69 (1.34–2.12)
Monthly Income				
No income	217 (35.3)	397 (64.7)	*Ref*	--
<10000 Shilling	215 (39.7)	327 (60.3)	1.12 (0.97–1.30)	
10000+ Shilling	374 (38.2)	606 (61.8)	1.08 (0.95–1.23)	
Sexual Position Preference				
Predominantly insertive	383 (38.7)	607 (61.3)	*Ref*	--
Predominantly receptive	165 (35.9)	295 (64.1)	0.93 (0.80–1.07)	
Receptive and insertive	258 (37.6)	428 (62.4)	0.97 (0.86–1.10)	
Age at first anal/oral sex with a man^c^				
<15 Years	73 (34.8)	137 (65.2)	*Ref*	*Ref*
15 to 17 Years	289 (40.0)	433 (60.0)	1.15 (0.94–1.41)	1.02 (0.84–1.24)
18+ Years	444 (36.9)	760 (63.1)	1.06 (0.87–1.30)	0.94 (0.78–1.14)
Number of different male sex partners (past 1 month)^*b*^				
<2	391 (40.9)	565 (59.1)	*Ref*	*Ref*
2+	415 (35.2)	765 (64.8)	0.86 (0.77–0.96)	0.92 (0.83–1.02)
Received money/gifts for sex (ever)				
No	325 (37.1)	551 (62.9)	*Ref*	--
Yes	481 (38.2)	779 (61.8)	1.03 (0.92–1.15)	
Condom use with last male sex partner^c^				
No	235 (35.2)	432 (64.8)	*Ref*	*Ref*
Yes	571 (38.9)	898 (61.1)	1.10 (0.98–1.25)	1.11 (0.99–1.24)
Contacted by peer/outreach worker in last 3 months^c^				
No	186 (23.4)	610 (76.6)	*Ref*	*Ref*
Yes	620 (46.3)	720 (53.7)	1.98 (1.72–2.27)	1.64 (1.43–1.87)

^a^Physical sites include hotspots, nightclubs, etc.; Virtual sites include social media, dating apps, etc.

^*b*^*a priori* chosen for fully-adjusted regression model.

^c^p < .10 in partial regression model (including Round 2 vs. 1 variable and present variable).

### Program exposure by HIVST kit use

**Fig 2:**
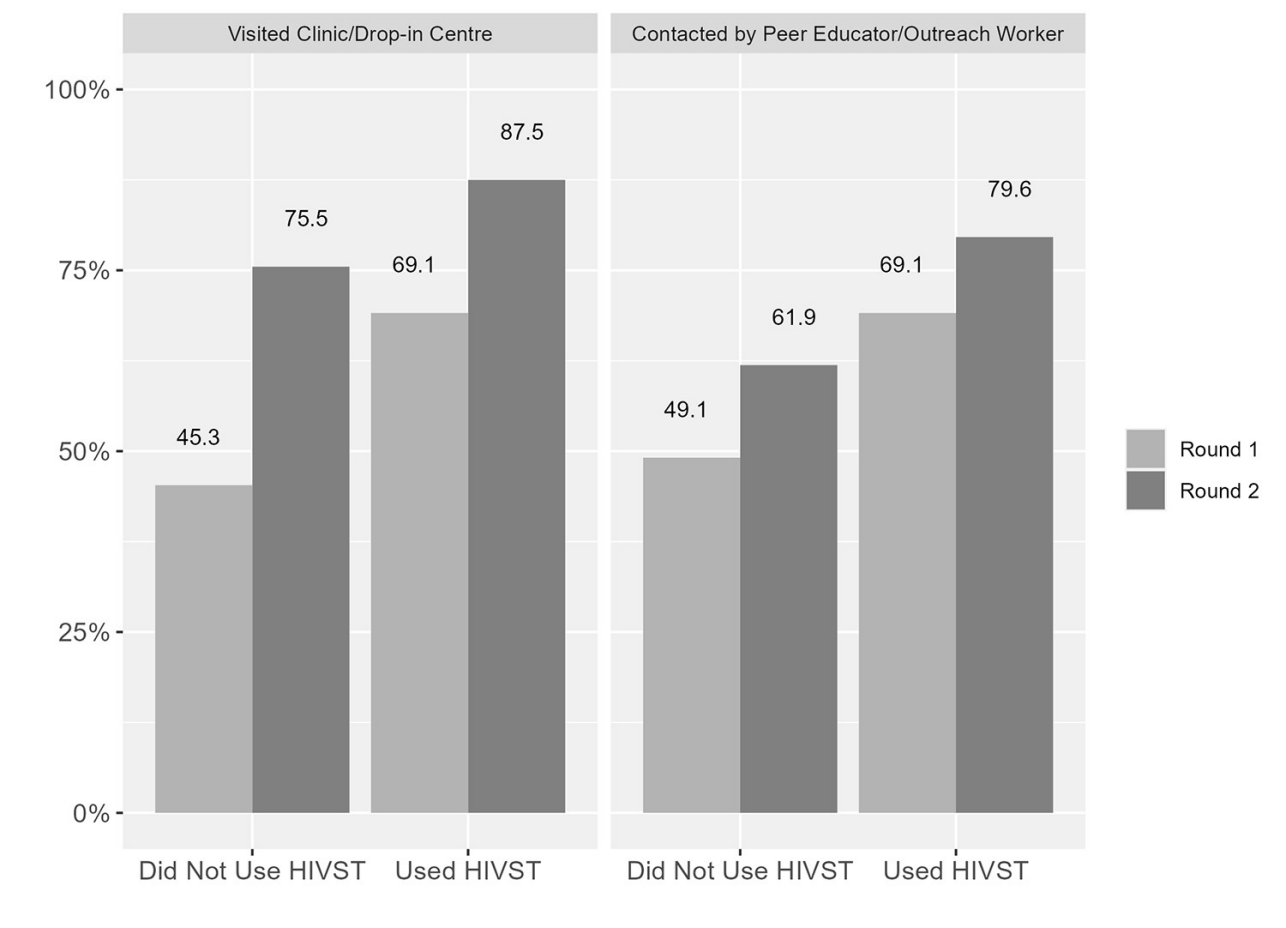
Contact/Visit exposure by type of exposure, round and lifetime use of HIV self-testing (HIVST) kits.

**Fig 3:**
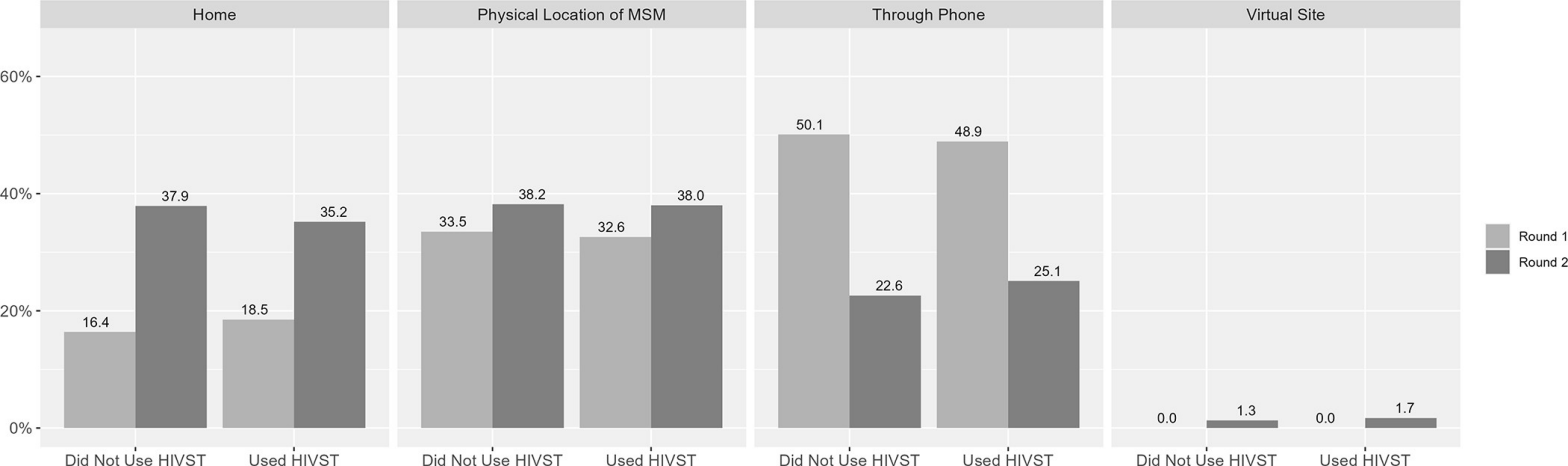
Contact/Visit exposure: “The last time you were contacted by a peer educator/outreach worker, how did he contact you?” by round and lifetime use of HIV self-testing (HIVST) kits.

**Fig 4:**
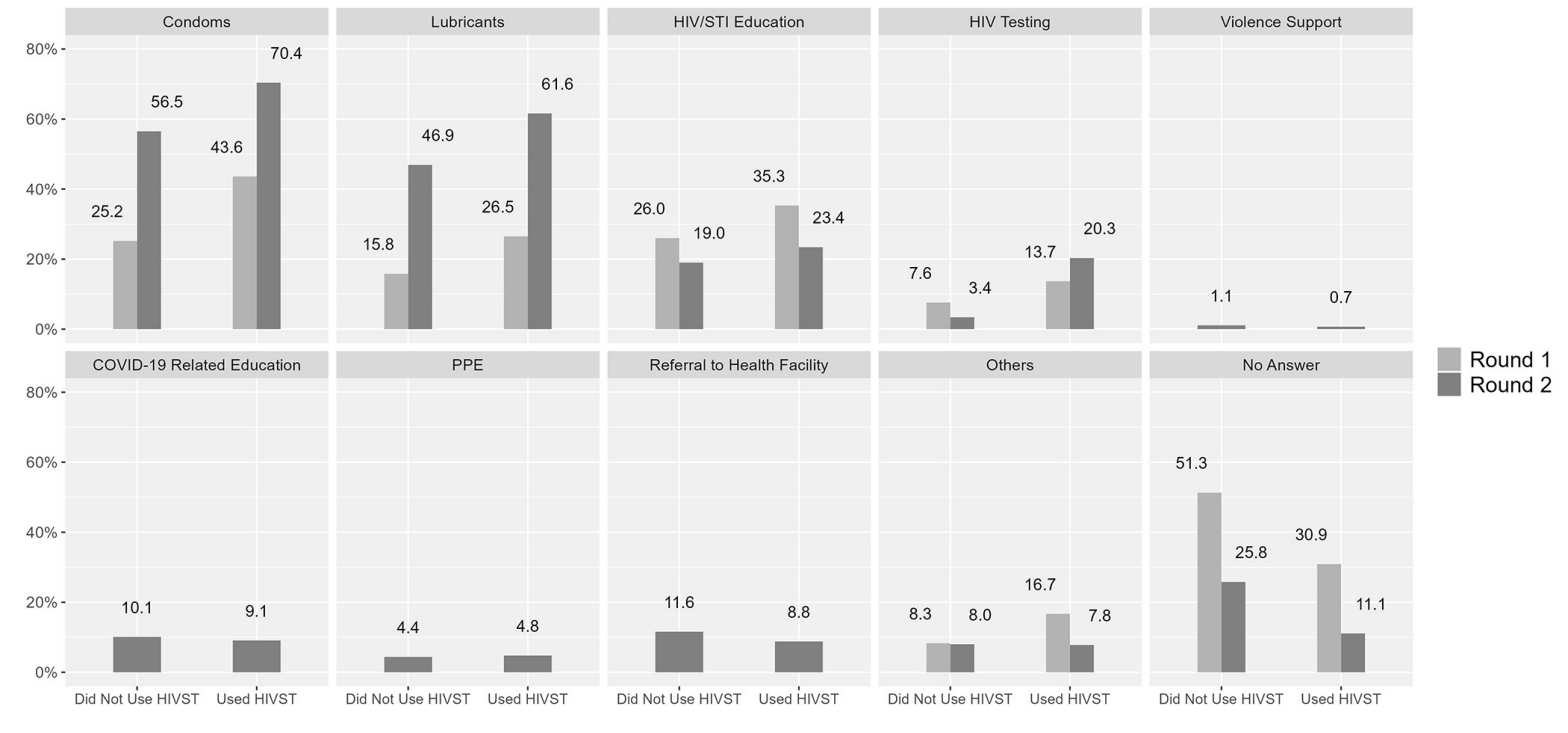
Service exposure: “The last time you were contacted by a peer educator/outreach worker, what services did you use?” by round and lifetime use of HIV self-testing (HIVST) kits.

**Fig 5:**
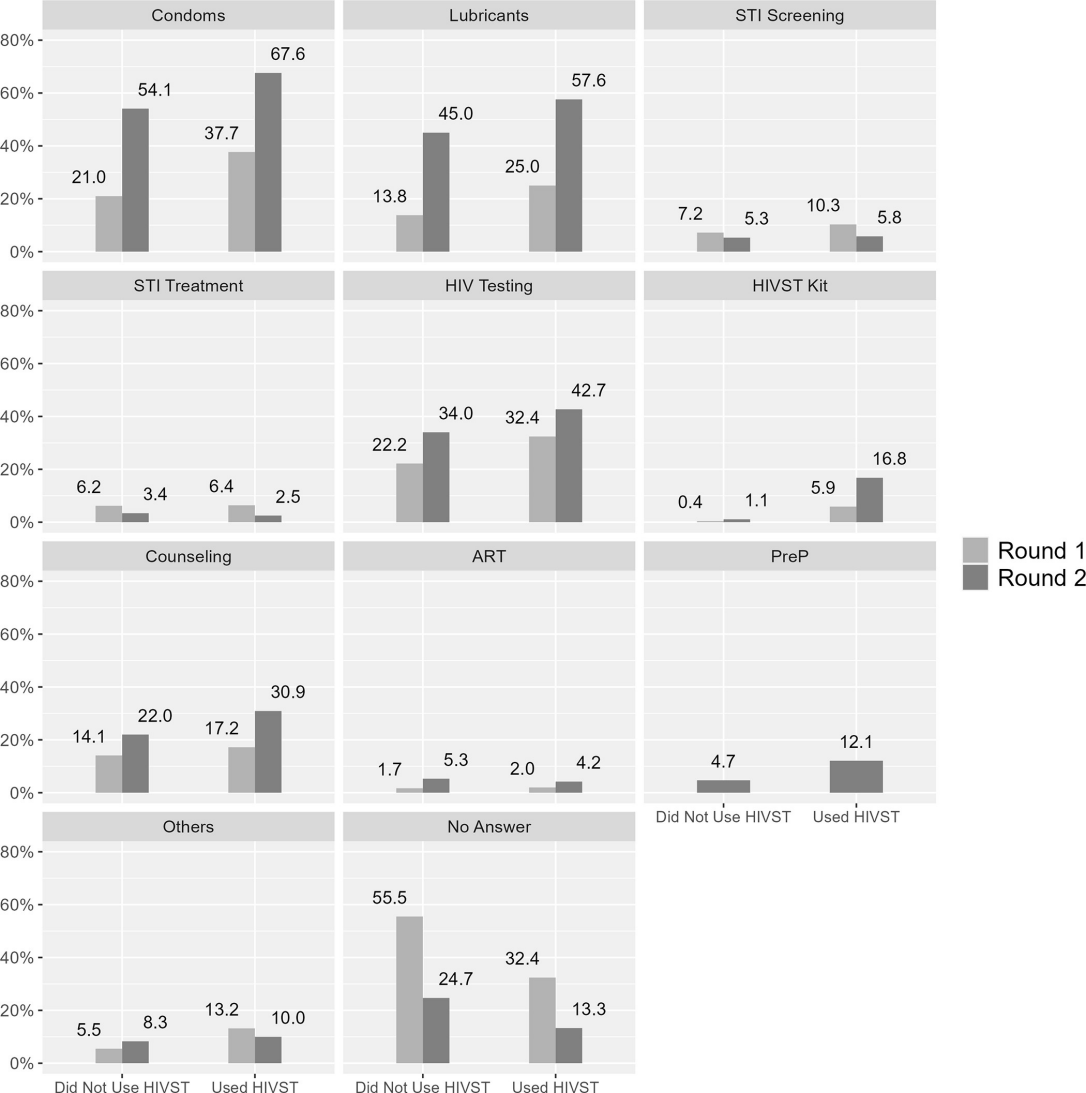
Service exposure: “The last time you visited a clinic or drop-in centre, what services did you use?” by round and lifetime use of HIV self-testing (HIVST) kits.

Figs 2 to 5 illustrate program exposure by HIVST kit use from both rounds of data collection. In terms of Contact/Visits, exposure was higher in men who reported HIVST kit use across both rounds. For example, 69% of those reporting HIVST kit use reported visiting a clinic/drop-in centre in the last 3 months, compared to 49% of men who did not report HIVST kit use in Round 1; in Round 2, the proportions were 88% and 76%, respectively (Fig 2).

Similarly, 69% of men ever using HIVST kits reported being contacted by a peer educator/outreach worker in the last 3 months, compared to 45% of men who did not use HIVST kits in Round 1, with the proportions being 80% for those using HIVST kits and 62% for those not using HIVST kits in Round 2 (Fig 2).

There were few differences between men who reported HIVST kit use and those who did not, when it came to how they were contacted by a peer educator (Fig 3).

In terms of services used at last contact with a peer educator, and among men reporting HIVST kit use, the proportion who reported receiving condoms (71% vs. 44%), lubricant (62% vs. 27%), and HIV testing increased from Round 1 to Round 2 (Fig 4). Of note, in Round 2, a minority of men reported receiving COVID-19 related education (~9% in either group) and personal protective equipment (~5% in either group) from peer educators/outreach workers.

Of some interest, irrespective of HIVST kit use, a smaller percentage of men reported using STI screening and STI treatment services in Round 2, compared to Round 1 (Fig 5) in clinical settings.

## Discussion

Compared to baseline, our results showed the proportion of MSM who had ever heard of HIVST increased from 75% to 94% at endline assessments, while the proportion of men reporting ever having used an HIVST kit increased from 20% to 53%. Education level was strongly associated with both having heard of HIVST and reporting its use. Highlighting the importance of community-based participatory methods, and thus the contribution of community-based MSM groups to our study [25,36], we found a strong association between both our outcomes and exposure to MSM-specific programs. For example, we found that 77% (n: 621/807) of men who reported using an HIVST kit reported also being contacted by a peer worker in the last 3 months, compared to 54% (n: 722/1,332) of men who did not report using an HIVST kit. Similarly, 65% (n: 1,277/1,977) of men who reported having heard of HIVST reported being contacted by a peer worker in the last 3 months, compared to 35% (n: 124/351) of men who reported they had never heard of HIVST. This was supported by our secondary analyses, as those reporting HIVST kit use were more likely to report program exposure. These results suggest that scaling up HIVST should include outreach components, most particularly in the form of participation from MSM-specific programs and community groups to achieve optimal levels of coverage and linkage to services. Of note are the general increases in program exposure from Round 1 to Round 2; from a program perspective it would be important to understand the specific mechanisms through which program exposure increased, to both men who did and did not report using HIVST kits, especially in light of the COVID-19 pandemic in Kenya. Although program data indicated an initial decrease in testing and outreach at the start of the pandemic in Kenya, CBOs quickly adapted to the imposition of non-pharmaceutical interventions used to mitigate the spread of COVID-19 by emphasizing safe clinical spaces (e.g., provision of masks, hand sanitizer, and an appointment-based system), the use of virtual platforms to connect to MSM, and the distribution of HIVST kits [24]. Although HIVST kits were made more available over the course of the pandemic, and could potentially explain the increased uptake of kits observed in the data, we feel strongly that community engagement helped generate demand for tests, and was key to informing community members of their availability. Our study was not designed to tease out the relative contributions of increased availability above and beyond community engagement; but this is an important area for future research.

To this end, research conducted in this context has demonstrated the impact of shifting outreach efforts to virtual platforms and working more systematically in physical sites, in concert with efforts to increase the agency of MSM, including provision of HIVST kits [24]. Embedding the project within a larger HIV program managed by CBOs likely contributed to both exposure to HIV-specific programming, and the increased uptake of HIVST kits. Although previous studies have shown high willingness and acceptability of HIVST in Kenya [37–41], and in light of our own results demonstrating high familiarity and uptake of HIVST after the completion of the HIVST project, other aspects of comprehensive HIV prevention/intervention programming should not be ignored, including the importance of acceptability and accessibility of programmes, and the impact that these aspects have on linkage, and retention to care [42]. Moreover, it has been recognized that HIVST should be just one tool available for HIV programmes, and considerations like cost [38,41], challenges with self-use [43], and equity need to be taken into account [44]. At an estimated unit cost of $8.68 USD for each kit, plus additional costs related to confirmatory testing [45], questions regarding best strategies (including appropriate population, tactics, and communication) to scale up testing availability to optimize cost-effectiveness remain [41,44,46,47]; although new initiatives have the potential to reduce cost per test to $1 USD [48]. The increased opportunity for engagement with MSM in offering new prevention technologies (like HIVST), the role that these technologies have in increasing agency of MSM, their cost-effectiveness, and the synergistic impact of community participation in comprehensive HIV programming should be future avenues of research.

Higher levels of education were found to be associated with HIVST use in a population-based study by Mwangi et al. in Kenya [49]; however, unlike our study, the authors found a positive association between number of partners and HIVST use. The reasons behind this discrepancy should be explored, although the study by Mwangi et al. was focused on the general Kenyan population, which may have contributed to the disparate findings. Regardless, our results indicate lower uptake of HIVST among MSM with less education, and who had a greater number of sexual partners in the past one month, suggesting that some segments of the MSM communities in the counties of our study may need more targeted efforts to bring HIVST use to scale. The reasons behind why those who exclusively used virtual sites and those who exclusively used physical sites were less likely to use HIVST kits, relative to those who used both, should also be explored in future research. Bhattacharjee et al. demonstrated that men who used both virtual and physical sites to meet their partners were most likely to be contacted by a peer educator or outreach worker, and were more likely to visit an MSM-focused clinic [3], suggesting higher levels of program exposure and access.

### Strengths & limitations

Our study had a number of strengths, including sampling men from both physical and virtual sites, biological data on HIV status, and the availability of baseline and endline data. Our study also had a number of important limitations. First, data were collected via face-to-face interviews, and were thus subject to social desirability bias, which may have resulted in underreporting of risky behaviours and/or HIV status, and overreporting of awareness and use of HIVST kits. Second, the sampling method included multi-stage sampling of physical and virtual sites, and recruitment of a limited number of contacts; this may have resulted in selection bias introducing a higher degree of homogeneity in samples. Third, questions regarding whether or not Round 2 respondents had participated in Round 1 were not included, and there was no attempt to screen out Round 1 participants; therefore there exists the possibility that Round 1 participants were included in Round 2, the degree to which cannot be estimated from our data. Because a probability-based sampling design was used and our goal was to estimate trends in time at the aggregate level (vs. examining changes in time at the individual level), the potential inclusion of Round 1 participants in Round 2 is justified, it is still important to note that if our sampling in Round 2 was biased towards inclusion of those who were more likely to have reported both knowledge and use of HIVST kits from Round 1, then we would also expect that we would observe an increase in both outcomes in Round 2. Indeed, this is what was observed; however, it should be noted that since we used a probability-based sampling frame, there was an equivocal potential to include participants who did not participate in Round 1. Moreover, descriptive statistics demonstrated that Round 2 participants differed from Round 1 participants by where they met their partners (i.e., in physical venues vs. virtual), and by education level (both of which were adjusted for in multivariable regression models), suggesting that there was a degree of change in participants between the two rounds, which the COVID-19 pandemic may have contributed to. Nevertheless, we cannot rule out the possibility that a substantial inclusion of Round 1 participants who reported knowledge and/or use of HIVST kits in Round 2 could be an alternative explanation for our results.

## Conclusions

We found a substantial increase in the proportion of men who had heard of HIVST, and who reported HIVST kit use between our baseline and endline surveys. The contribution of community-based methods to the high uptake observed in our study should be explored. Although study findings are encouraging, further work to engage specific MSM subpopulations, such as those with fewer years of education, may result in more widespread use of HIVST among MSM in Kenya.

## Supporting information

S1 Checklist Inclusivity in global research. CBO: Community-Based Organization. HAPA Kenya: HIV & AIDS People’s Alliance of Kenya. HOYMAS: Healthy Options for Young Men on HIV/AIDS/STIs. MAAYGO: Men Against AIDS Youth Group Organization. MPEG: Mamboleo Peer Empowerment Group. MSM: Men who have sex with men. NASCOP: National AIDS and STI Control Programme. PHDA: Partners for Health and Development in Africa.
(DOCX)

S1 Table Crude and fully-adjusted prevalence ratios (PR) and 95% confidence intervals (95% CI), multivariable regression models, association of sample rounds (Round 1 vs. Round 2) on HIV self-testing outcomes, stratified by education level, Kenya HIV self-testing study. PR: Prevalence Ratio. CI: Confidence Interval. ^a^Adjusted for places where male partners met, county, age, number of different male sex partners (past 1 month), received money/gifts for sex (ever), condom use with last male sex partner, and contacted by peer/outreach worker in the last 3 months. ^b^Adjusted for places where male partners met, county, age, age at first anal/oral sex with a man, number of different male sex partners (past 1 month), condom use with last male sex partner, and contacted by peer/outreach worker in the last 3 months.
(DOCX)
