# Supplementary material for: Assessing awareness and use of HIV self-testing kits after the introduction of a community-based HIV self-testing programme among men who have sex with men in Kenya
Source: PLOS Glob Public Health. 2023 Aug 18;3(8):e0001547. doi: 10.1371/journal.pgph.0001547 (PMC10437899; doi:10.1371/journal.pgph.0001547)
Supplement: S1 Checklist — CBO: Community-Based Organization. HAPA Kenya: HIV & AIDS People’s Alliance of Kenya. HOYMAS: Healthy Options for Young Men on HIV/AIDS/STIs. MAAYGO: Men Against AIDS Youth Group Organization. MPEG: Mamboleo Peer Empowerment Group. MSM: Men who have sex with men. NASCOP: National AIDS and STI Control Programme. PHDA: Partners for Health and Development in Africa. (DOCX) [file pgph.0001547.s001.docx]

Inclusivity in global research

PLOS’ policy on inclusivity in global research aims to improve transparency in the reporting of research performed outside of researchers’ own country or community and ensures that PLOS publications reporting global research adhere to high standards for research ethics and authorship. Authors of relevant research articles may be asked to complete the questionnaire below, which outlines ethical, cultural, and scientific considerations specific to inclusivity in global research. This questionnaire may be requested when researchers have travelled to a different country to conduct research, if research uses samples collected in another country, research with Indigenous populations or their lands, or if research is on cultural artefacts. Researchers travelling to another country solely to use laboratory equipment will not normally be required to complete the questionnaire. However, the questionnaire can be requested at the journal’s discretion for any submission – if you have been requested to complete this questionnaire by the PLOS journal you submitted to, please do so.

Please complete the questionnaire below and include this as a Supporting Information file with your manuscript. Note that if your paper is accepted for publication, this checklist will be published with your article in the supporting information files. Please ensure that you reference the checklist in the main body of your manuscript. We suggest adding a subsection ‘Inclusivity in global research’ to your Methods section and adding the following sentence: “Additional information regarding the ethical, cultural, and scientific considerations specific to inclusivity in global research is included in the Supporting Information (SX Checklist)”

The questions have been designed to be applicable to a wide range of study types, and there are subsections for both human subjects research and non-human subjects research. If any of the questions are not relevant to your research please mark them as “N/A” as appropriate.

**Ethical considerations, permits and authorship**

*This section is applicable to all research types.*

Provide details as to who granted permissions and/or consent for the study to take place in the Methods section of your manuscript. This should include the names of **all** ethics boards, governmental organizations, community leaders or other bodies that provided approval for the study. If individuals provided approval refer to these people by their role or title but do not list their name(s).

Reported on page number: 5

“Ethics approval was obtained from the institutional review boards of the University of Nairobi (P557/08/2018) and University of Manitoba (HS22205). We followed NASCOP’s guidelines in conducting sexual and reproductive health research with adolescent key populations whereby those 15 years and above are considered mature/emancipated minors. Given this, and the fact that HIV testing in Kenya without a guardian is 15 years and above, ethics boards in Kenya and Manitoba allowed respondents to give consent and participate in the study without guardian consent.”

If there were any deviations from the study protocol after approval was obtained please provide details of these changes in the Methods section of your manuscript.
Did this study involve local collaborators that are residents of the country where the research was conducted or members of the community studied? If you do not have any authors from said communities, please provide an explanation for this below.

Yes, the study involved local collaborators who are residents of Kenya, and are members of the community under study. The study included broad representation from MSM groups such as MAAYGO (Men Against AIDS Youth Group Organization), HOYMAS (Healthy Options for Young Men on HIV/AIDS/STIs), Ishtar MSM, HIV and AIDS People’s Alliance of Kenya, and Mamboleo Peer Empowerment Group.

Reported on page number: No deviations

Everyone listed as an author should meet PLOS’ criteria for authorship and all individuals who meet these criteria should be included in the author byline, rather than the acknowledgements. Authorship criteria is based on the International Committee of Medical Journal Editors (ICMJE) Uniform Requirements for Manuscripts Submitted to Biomedical Journals - for further information please see here: <https://journals.plos.org/plosone/s/authorship>.

**Human subjects research (e.g. health research, medical research, cross-cultural psychology)**

Did you obtain written informed consent from a representative of the local community or region before the research took place? How did you establish who speaks for the community? Details of written informed consent obtained from study participants should be reported separately in the Methods section of your manuscript.

We did not obtain written informed consent from representatives of the local community or region, although ethics approvals were obtained from both the University of Nairobi and University of Manitoba. However, the University of Manitoba has had a decades-long presence in Kenya, and continues to have longstanding relationships with community-based organizations across Kenya, including in the three study sites of this project. The study was implemented by the University of Manitoba and Partners for Health and Development in Africa (PHDA), in partnership with the National AIDS and STI Control Programme (NASCOP), as well as community-based partners G10 (an MSM research network in Kenya), and three community-based organizations (CBOs) in Kenya: Mamboleo Peer Empowerment Group (MPEG in Kiambu), Men Against AIDS Youth Group (MAAYGO in Kisumu), and the HIV & AIDS People’s Alliance of Kenya (HAPA Kenya in Mombasa). These groups, and others, were consulted prior to the start of the study, and helped co-design the study, including the intervention itself.

How did members of the local community provide input on the aims of the research investigation, its methodology, and its anticipated outcome(s)?

We took a community-based research methods approach in which the study was co-designed with community researchers and community-based organizations from all three study counties in Kenya. Community members were involved in all aspects of the study design (including reviewing and finalizing data collection tools), and some community researchers were hired to help with recruitment, and aspects of the interviewing.

When engaging with the local community, how did you ensure that the informed consent documents and other materials could be understood by local stakeholders?

Community members helped design all documents, and were instrumental in translation of documents to Kiswahili. Participants were given the option of answering the survey in English or Kiswahili.

Will the findings of the research be made available in an understandable format to stakeholders in the community where the study was conducted (e.g. via a presentation, summary report, copies of publications, etc.)? Please provide details of how this will be achieved.

Yes. Findings, in the form of short reports, presentations, and focus group discussions, have been disseminated to community organizations involved with the study. Some community members have been included as co-authors on manuscripts produced as a result of the study, including the present manuscript. As per our study protocol: “the results of this study will be disseminated through the Key Population Technical Working Group and the HIV Testing Services Technical Working Group led by NASCOP at both national and county level at regular intervals, as each step of the study is completed. The findings will be also shared with G10 members who are part of the MSM CBOs representing different regions of Kenya. Community meetings will be organized at the study sites to share the findings with MSM community members.”

**Non-human subjects research using specimens/ animals collected as part of the study, or those housed in archival collections. Examples include archaeology, paleontology, botany and zoology.**

Did the permission you obtained from a local authority to perform the study include an agreement on access to outputs and benefit sharing? This may include procedures to enable fair distribution of the benefits and resources arising from the research performed. Please include any details of Prior Informed Consent and Benefit Sharing Agreements obtained. These may be required by field-specific regulations, for example the Convention on Biological Diversity (CBD) and the associated Nagoya Protocol.

If the material used in your study was imported, please A) provide the year it was imported and B) indicate whether permits were obtained to import/export the materials used, C) provide details of any permits obtained. If this information is not available, please indicate this.

If you used archival specimens, please state how the material used in your study was acquired by the institute it is held in and provide details of any permits obtained for the original excavations/ sample collection. If this information is not available, please indicate this.

How was the potential cultural significance of the materials collected in your study to local communities considered in your research design? Were Indigenous peoples and/or local researchers and institutions involved with archaeological excavations / collection of specimens? If so, please provide a description of their involvement.

If your manuscript includes photographs of human remains please indicate whether authors obtained permission from descendants or affiliated cultural communities to do so.
