# Supplementary material for: Assessing awareness and use of HIV self-testing kits after the introduction of a community-based HIV self-testing programme among men who have sex with men in Kenya
Source: PLOS Glob Public Health. 2023 Aug 18;3(8):e0001547. doi: 10.1371/journal.pgph.0001547 (PMC10437899; doi:10.1371/journal.pgph.0001547)
Supplement: S1 Table — CI: Confidence Interval. aAdjusted for places where male partners met, county, age, number of different male sex partners (past 1 month), received money/gifts for sex (ever), condom use with last male sex partner, and contacted by peer/outreach worker in the last 3 months. bAdjusted for places where male partners met, county, age, age at first anal/oral sex with a man, number of different male sex partners (past 1 month), condom use with last male sex partner, and contacted by peer/outreach worker in the last 3 months. (DOCX) [file pgph.0001547.s002.docx]

|  |  | **Have you heard of HIV self-testing?^a^ (N: 2,328)** | | **Crude PR**  **(95% CI)** | **Adjusted PR**  **(95% CI)** |
| --- | --- | --- | --- | --- | --- |
| **Education Level** |  | **Yes (No., %)** | **No (No., %)** |  |  |
| Up to Primary | Round (row %) |  |  |  |  |
|  | Round 1 | 116 (53.0) | 103 (47.0) | *Ref* | *Ref* |
|  | Round 2 | 110 (85.9) | 18 (14.1) | 1.62 (1.41-1.87) | 1.48 (1.12-1.95) |
| Up to Secondary | Round (row %) |  |  |  |  |
|  | Round 1 | 423 (76.8) | 128 (23.2) | *Ref* | *Ref* |
|  | Round 2 | 487 (93.5) | 34 (6.5) | 1.22 (1.16-1.28) | 1.20 (1.05-1.38) |
| Post-Secondary | Round (row %) |  |  |  |  |
|  | Round 1 | 304 (85.9) | 50 (14.1) | *Ref* | *Ref* |
|  | Round 2 | 537 (96.8) | 18 (3.2) | 1.13 (1.08-1.18) | 1.13 (0.97-1.30) |

|  |  | **Have you taken an HIV self-test?^b^ (N: 2,136)** | | **Crude PR**  **(95% CI)** | **Adjusted PR**  **(95% CI)** |
| --- | --- | --- | --- | --- | --- |
| **Education Level** |  | **Yes (No., %)** | **No (No., %)** |  |  |
| Up to Primary | Round (row %) |  |  |  |  |
|  | Round 1 | 24 (14.0) | 147 (86.0) | *Ref* | *Ref* |
|  | Round 2 | 38 (34.9) | 71 (65.1) | 2.48 (1.58-3.90) | 2.15 (1.24-3.73) |
| Up to Secondary | Round (row %) |  |  |  |  |
|  | Round 1 | 89 (17.7) | 413 (82.3) | *Ref* | *Ref* |
|  | Round 2 | 237 (48.8) | 249 (51.2) | 2.75 (2.23-3.39) | 2.50 (1.95-3.20) |
| Post-Secondary | Round (row %) |  |  |  |  |
|  | Round 1 | 91 (27.3) | 243 (72.8) | *Ref* | *Ref* |
|  | Round 2 | 327 (61.2) | 207 (38.8) | 2.25 (1.86-2.71) | 2.16 (1.70-2.74) |
